# Maternal Perceptions of Infant Behavior as a Potential Indicator of Parents or Infants in Need of Additional Support and Intervention

**DOI:** 10.3389/fpubh.2021.630201

**Published:** 2021-10-20

**Authors:** Leslie A. Frankel, Tomotaka Umemura, Kendall A. Pfeffer, Elisabeth M. Powell, K. R. Hughes

**Affiliations:** ^1^Department of Psychological, Health and Learning Sciences, University of Houston, Houston, TX, United States; ^2^Department of Psychology, Hiroshima University, Hiroshima, Japan; ^3^Department of Psychology, New School for Social Research, New York, NY, United States; ^4^Western Psychological and Counseling Services, Vancouver, WA, United States

**Keywords:** infant behavior, parenting, infant risk, infant-parent bonding, parent-child bonding

## Abstract

The goal of the present study is to examine the relationship between early infant behaviors, which can be easily reported by parents, with parent-infant bonding and maternal mental health. It has long been established that child characteristics and behaviors have a significant impact on parent well-being and how parents respond to their infants. Examining parent perceptions of challenging infant behaviors may help health professionals identify high risk infants in need of intervention and mothers in need of additional support. Mothers of 73 infants between the ages of 3.5 weeks and 6 months filled out questionnaires. Infant stomach issues were positively correlated with bonding issues, maternal anxiety and maternal depression. Infant crying issues were also positively correlated with bonding issues, maternal anxiety and maternal depression. Potential clinical and research applications of the instrument include early identification of caregivers in need of support and screening for further clinical assessment and care.

## Introduction

Mothers are most often the primary caretakers for their young infants, yet little is known about how they perceive common challenging behaviors. It has been established that child characteristics and behaviors have a significant impact on parent well-being and, consequently, on how parents respond to their infants (1–3). Currently, hospital and community-based interventions target parents who are deemed to be at-risk due to specific risk factors such as parental age and rarely rely on parent perception of infant behavior to triage parents for professional help or intervention. Examining parent perceptions of challenging infant behaviors may help health professionals identify high risk infants in need of intervention and parents in need of additional support. Such efforts are needed to decrease the adverse impact of normative stressors on parent-child relationships and so that interventions can be more targeted, cost-effective, efficient and potentially more beneficial to the parent-child relationship, attachment quality and later infant outcomes.

Early infant behavior is important because it relates to infant-parent attachment and is predictive of behavior throughout life (4). Research evidence suggests that infant behaviors are related to maternal risk of postpartum depression. To this point, qualities of the infant, such as prolonged, excessive or inconsolable crying (5, 6) increase a mother's risk of postpartum depression, something that impacts roughly 14.5 percent of new mothers within the first 14.5 months of giving birth (7) and has adverse effects on the parent-child relationship (8). Maternal mental health can also impact infant behavior. To this point, postpartum depression impacts infant development across a variety of domains including motor development and cognitive development (9).

Difficulties with infant feeding are highly correlated with crying and are often encompassed within the working definition of an infant with “colic” (10). Less frequently studied infant behaviors that are nonetheless encompassed in many parents' working definition of a “difficult” infant include how good of a sleeper and eater the infant is. Infants who have difficulty with these early self-regulatory behaviors (i.e., crying, sleeping and feeding) are at risk for adverse outcomes later in life (11), suggesting the need for further research into these behaviors. To this point, the volume and timing of infant sleep is one of the most important infant behaviors to new parents (12). However, the above outlined behaviors do not occur in isolation, and each may impact multiple areas of functioning. For example, feeding an infant at night has been shown to create sleeping problems (13–15), and sleeping problems have been associated with stomach aches in young children (16). Stomach issues are noted in research as one of the leading reasons that new parents take children to the doctor outside of routine visits (17). Taken together, these early infant behaviors (i.e., crying, eating, sleeping and stomach issues) are areas of both explicit and implicit concern for parents.

Infant crying is a normative behavior that has been found on average to occur for just over 2 hours per day for infants between 1 and 3 months and about 1 hour per day for infants between 4 and 6 months (18). Concern over infant crying emerges when the crying is excessive as it is associated with maternal depression (19) and increased risk of harm to the infant. For example, infant crying has been implicated as a behavior that could provoke infant shaking by parents (20), increasing risk for injuries such as Shaken Baby Syndrome (SBS).

It is important for clinicians to quickly gather information from parents about their areas of concern related to their infant's behavior.

Existing interventions in the newborn period might benefit from more specified targeting within the population level that is similar to the screenings that are currently implemented in the area of postpartum depression—with assessments that quickly gather information and triage parents to more support. The purpose of this study is to demonstrate that parental perceptions of infant behavior are related to parent bonding and parent well-being. Our hypothesis is that parental belief that their child is having trouble in an area such as crying, eating, sleeping and stomach issues will be correlated with worse outcomes such as impaired bonding or heightened parental depression or anxiety.

## Materials and Methods

### Participants

Seventy-three mothers of infants between the age of 3.5 weeks and 6 months (characteristics of the sample outlined in Table 1) were included in the study. Parents filled out questionnaires on anxiety, depression, infant bonding and infant behaviors as part of a larger cross-sectional study investigating the impact of participation in infant floating classes on parent mental health, bonding and infant behavior. Infant floating classes involve placing a flotation device around an infant's neck so that the infant can float in a tub of water and kick their feet. There were no significant differences in any measures across whether parents participated in floating classes with their infants; however, whether or not parents participated in floating classes is included in all analyses to control for any effect of group status on results. The sample was originally 74 parents, but only one father participated in the study. Due to the small sample of fathers, the father was dropped from the sample.

**Table 1:** Demographics of sample (*n* = 73).

**Variables**	**% (SD)**
**Parent Sex**	
Female	73
Parent Age	32.03 (4.51)
**Child Sex**	
Male	43
Female	31
Child age in months	3.65 (1.55)
**Race**	
White	54
African American	3
Asian	8
Multi-racial	6
Not reported	2
Hispanic/Non-hispanic	15/58
**Relationship Status**	
Single, never married	3
Married	65
Separated	1
Relationship, living together	4
**Yearly Household Income**	
Less than $50,000	14
$50,000–100,000	10
Above $100,000	49
**Highest Education**	
High school	2
Some college, no degree	15
College degree	31
Advanced degree	25

### Procedures

Participants were recruited through a local infant floating facility in Houston, TX as well as through online postings on Facebook. Participants completed initial questions to confirm eligibility online, including being a parent of an infant between the ages of 3.5 weeks and 6 months old. Participants were prescreened and excluded from the study if they self-reported that infant had any known health issues that might interfere with the child's physical abilities. Four participants were excluded based on this prescreening. Participants were informed that they would be entered into a raffle to win three free sessions at the infant aquatic therapy facility as compensation for their participation, and informed consent was obtained through Qualtrics prior to any study activities. This study was approved by the Institutional Review Board at the University of Houston.

### Measures

#### The Baby Actions and Behavior Index

The Baby Actions and Behavior Index (BABI) infant behavior scale measures critical domains of infant behavior: eating issues, stomach issues, crying, and sleep issues. The authors (LF and EP) created 14 questions assessing infant behavior in these domains: eating (“How satisfied are you with the amount your baby eats”), stomach issues (e.g.,” Compared to other babies your infant's age, how much gas does your baby have?”), crying (e.g., “How much does your baby cry?”), and sleep issues (e.g., “Compared to other babies your infant's age, how often does your baby wake up in the middle of the night?”) (see Table 2 for full scale).

**Table 2:** Original items in BABI infant behavior scale.

**Cry Items**	
Cry_1_Reversed	How much does your baby cry?
Cry_2_Reversed	How often does your baby cry?
Cry_3_Reversed	How intensely does your baby cry?
Cry_4_Reversed	How easily is your infant comforted by you when he/she cries?
**Eat Items**	
Eat_1_Reversed	How satisfied are you with the amount your baby eats?
Eat_2_Reversed	How satisfied are you with how often your baby eats?
Eat_3_Reversed	Compared to other babies your infant's age, how much does your baby eat?
**Sleep Items**	
Sleep_1	How satisfied are you with your infant's sleep?
Sleep_2_Reversed	Compared to other babies your infant's age, how much does your baby sleep?
Sleep_3	Compared to other babies your infant's age, how often does your baby wake up in the middle of the night?
Sleep_4_Reversed	Compared to other babies your infant's age, how much does your baby nap?
**Stomach Items**	
Stomach_1	Compared to other babies your infant's age, how much reflux does your baby have?
Stomach_2	Compared to other babies your infant's age, how much gas does your baby have?
Stomach_3	Compared to other babies your infant's age, how much stomach pain does your baby have?

*The eating subscale was dropped in its entirety due to issues with item normality and low inter-item correlation. Sleep_4_Reversed was removed after EFA and CFA due to low correlation with other items and lack of face validity with other items*.

#### Mother-Infant Bonding Scale

Mother-Infant Bonding Scale [MIBS; (21)] is an eight-item, self-report scale assessing emotions mothers may have experienced toward their infants. This measure was designed to identify difficulties experienced by new mothers in establishing a relationship with their babies, and was intended for use in the first weeks after the child's birth through 4 months postpartum (22). Participants were asked to identify to what degree they have felt various emotions toward their infant in the past few weeks by responding on a four-point Likert scale from *very much (0)* to *not at all (3)*. Emotion prompts include “loving” [very much (0) to not at all (3)] and “resentful” [reverse scored; very much (3) and not at all (0)]; higher total scale scores indicate worse bonding. This measure has demonstrated high sensitivity in detecting bonding alterations between new mothers and their babies (23) and has evidenced moderate concurrent validity with two other measures: the Postpartum Bonding Questionnaire (24) and the Maternal Postpartum Attachment Scale (25). Reliability analyses have demonstrated a Cronbach's alpha, or internal consistency score of 0.71, evidencing acceptable reliability (21). The Cronbach's alpha for the MIBS was 0.59 in the present study.

#### Edinburgh Postnatal Depression Scale

Edinburgh Postnatal Depression Scale [EPDS; (26)] is a 10-item, self-report measure developed to help identify new mothers who may be at risk for postpartum depression. Research has demonstrated a potential long-term negative impact of postpartum depression on the child, including behavioral disturbances (27) and later cognitive deficits (28). This scale was created in response to research confirming that the period after childbirth is frequently characterized by some form of psychological distress for new mothers (29), and that at least 10–15% of mothers experience depression during this time (26). Participants respond to prompts about how they have felt in the past seven days on a four-point Likert scale with scores from 0 to 3 (e.g., *yes, all the time* to *no, not at all*; *yes, quite often* to *never*, etc.), and total scores range from 0 to 30. Higher scores on this scale indicate higher symptomology of depression. Item examples include “I have felt happy,” “I have felt sad or miserable,” and “things have been getting on top of me.” Cutoff scores have previously been determined; Cox, Holden and Sagovsky identified a cutoff of 12/13 for moderate depression, which was replicated by Harris (30) (using Diagnostic and Statistical Manual of Mental Disorders-III criteria [DSM-III]) and Murray and Carothers (31) (using Research Diagnostic Criteria [RDC]). Additionally, Cox, Holden and Sagovsky recommended a cutoff score of 10 to include minor depression as well as increased sensitivity of the scale or ability to capture people with a diagnosis of depression (32). This was also confirmed by Harris (30), as well as Murray and Carothers. Previous research using a cutoff of 10 has demonstrated accurate later classification of mothers at 4 weeks later (85.4% accurately classified) and 8 weeks later (82.5% accurately classified) (33). This measure has also demonstrated satisfactory validity, split-half reliability and adequate sensitivity to changes in depression over time (26). Reliability analyses have demonstrated a Cronbach's alpha of 0.87, evidencing good reliability (26); the Cronbach's alpha in the present study was found to be 0.87.

#### Generalized Anxiety Disorder

Generalized Anxiety Disorder is a seven-item scale (GAD-7; 33) assessing anxiety. This measure asks participants to identify how often they have been bothered by certain problems, and participants respond on a four-point Likert scale (with scores from 0 to 3) from *not at all* to *nearly every day*. Items include “feeling nervous, anxious, or on edge” and “not being able to stop or control worrying.” Scores range from 0 to 21 and higher scores on this scale indicate higher symptomology of anxiety. Scale authors suggest a cutoff point of 10 for identifying anxiety. At this cutoff, the scale has yielded a sensitivity of 89% in a primary care sample (34), and in a sample of pregnant and postpartum women, it has yielded a sensitivity of 76.0% (35). Reliability analyses evidenced a Cronbach's alpha of 0.92, demonstrating excellent reliability (34) and a Cronbach's alpha 0.91 in the present study. Additionally, this measure has been found to have found good construct as well as factorial and procedural validity (34).

### Data Analysis Plan

Exploratory factor analysis (EFA) and confirmatory factor analysis (CFA) were conducted to examine the validity of subscales in the BABI scale (36). This was done to ensure that the intended scales functioned as originally conceptualized before proceeding to any other analyses with the subscales. To explore our hypothesis that parental belief that their child is having trouble in an area such as crying, eating, sleeping and stomach issues will be correlated with worse outcomes such as impaired bonding or heightened parental depression or anxiety, correlations were conducted to examine the relationship between the BABI scale and important variables such as Mother-Infant Bonding, postpartum depression and anxiety. Variables were deleted listwise. Because the infants in our sample varied by almost 6 months in age, Cronbach's alphas were examined separately for all measures.

## Results

### Exploratory and Confirmatory Factor Analyses

Prior to use of the BABI to explore our hypothesis, preliminary construct validity was established with an exploratory factor analysis (EFA) and confirmatory factor analysis (CFA) using the Mplus statistical software [version 7.11, (37)] to ensure that subscales of the BABI measured their intended facets of infant behavior. Before conducting factor analyses, we checked the normal distribution of our items and their correlations. We found that all items were normally distributed, except for one item for eating issues, which slightly violated the normality assumption *EAT_1R*; skewness = 1.40 and Kurtosis = 1.96). In addition, the correlation between the other two items for eating issues (*EAT_2R* and *EAT_3R*) was unexpectedly small, *r* = 0.16. Due to these problems pertaining to items on eating issues, we decided to remove all three items for eating issues. There were three remaining scales: crying, sleeping and stomach issues. We also removed one item on sleeping issues (*SLEEP_4R*) due to its low correlations with other sleep items (*r* = *0.1*3 with *SLEEP_1* and *r* = −0.04 with *SLEEP_3*), possibly due to the fact that (*SLEEP_4R*) asks about infant napping whereas the other items (*SLEEP_1, SLEEP_2R*, and *SLEEP_3*) ask about general sleep behaviors. Low correlations among items led us to have a poor performance of a factor analysis (38). In the final analysis, we included 10 items: four items addressing crying issues (*CRY_1R, CRY_2R, CRY_3R*, and *CRY_4R*), three items addressing sleeping issues (*SLEEP_1, SLEEP_2R*, and *SLEEP_3*), and three items addressing stomach issues (*STOMACH_1, STOMACH_2*, and *STOMACH_3*).

We conducted EFA with the varimax rotation. The scree plot suggests that a three-factor model best captures the items in our scale. Additionally, the eigenvalue of the three-factor model was higher than one, whereas the eigenvalue of the four-factor model was less than one, further indicating the validity of the three-factor model. Factor loadings of the final three-factor model are presented in Table 3.

**Table 3:** Varimax-rotated factor loadings of exploratory factor analysis for the baby actions and behavior index (BABI).

	**Factor 1**	**Factor 2**	**Factor 3**
CRY_1R	0.89	0.07	0.10
CRY_2R	0.92	0.08	0.07
CRY_3R	0.81	0.13	−0.16
CRY_4R	0.66	−0.02	0.12
SLEEP_1	0.03	0.84	0.20
SLEEP_2R	0.18	0.86	−0.05
SLEEP_3	−0.00	0.82	0.05
STOMACH_1	0.11	0.20	0.68
STOMACH_2	−0.12	0.05	0.88
STOMACH_3	0.14	−0.06	0.90

**Figure 1:**
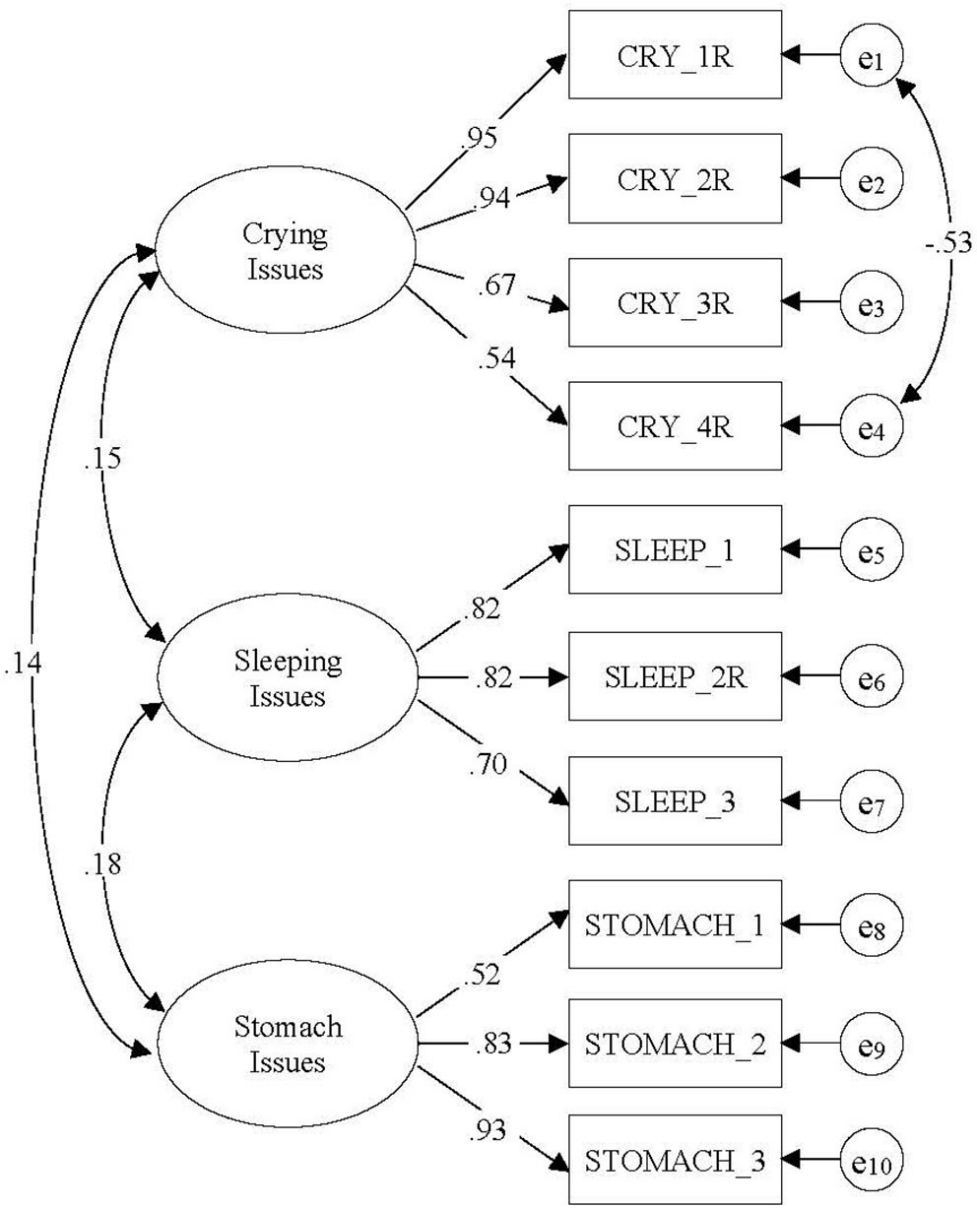
The confirmatory factor analysis model with crying issues, sleeping issues, and stomach issues. Numbers indicate standardized factor loadings and correction coefficient. CFI = 0.970, RMSEA = 0.068, and χ^2^_(df)_ = 41.47 (31), *p* = 0.099.

Due to the fact that the items were written as part of a subscale structure when the scale was originally conceptualized, we also conducted a CFA. Our CFA model result is presented in Figure 1. We included one correlation between residual variances for *CRY_1R* and *CRY_4R* because the modification index suggested the correlation. Furthermore, we believe that these two items are meaningfully related—infants who are harder to console when they cry will exhibit prolonged crying (i.e., cry more than other babies of the same age). The model fit was very good: *CFI* = 0.970, *RMSEA* = 0.068, and χ^2^_(df)_ = 41.47_(31)_, *p* = 0.099, suggesting that our scale consists of three distinct behavioral issues found in infants. Due to this very good model fit, we did not further conduct a *post-hoc* analysis to improve our model fit.

### Partial Correlations

Partial correlations between the BABI stomach issues, crying issues and sleep issues subscales and the Mother-to-Infant Bonding scale, anxiety scale, and postpartum depression scale were conducted in order to further establish construct validity (controlling for whether or not the mothers participated in floating class). Our hypothesis, that maternal belief of their child having trouble in an area such as crying, sleeping and stomach issues (*note: eating removed due to violation of the normality assumption*) will be correlated with worse outcomes such as impaired bonding or heightened parental depression or anxiety, was partially supported. Significant results were found for the following relationships: infant stomach issues were positively correlated with bonding issues, maternal anxiety and maternal depression; infant crying issues were also positively correlated with all three outcome variables: bonding issues, maternal anxiety and maternal depression (see Table 4).

**Table 4:** Partial correlation coefficients among study variables.

**Measure**	**MIBS**	**GAD anxiety**	**EPDS depression**
Infant stomach issues	0.31^**^	0.24^*^	0.25^*^
Infant crying issues	0.34^**^	0.26^*^	0.25^*^
Infant sleep issues	0.15	−0.03	−0.04

* *Correlation is significant at the 0.05 level*,

** *Correlation is significant at the 0.01 level (2-tailed). Controlling for whether the child had ever participated in floating classes. Missing variables deleted listwise. N = 69*.

### Cronbach's Alphas

Cronbach's alphas for the BABI were as follows: stomach issues was α = 0.77, crying issues was α = 0.86 and sleep issues was α =0.80.

## Discussion

In this study, perceived stomach issues and crying were both related to mother-infant bonding issues, higher maternal anxiety, and higher postpartum depression. Correlation coefficients are a common indicator of effect size. Although the correlations are statistically significant, they have relatively small effect sizes (39).

Assessments of newborns and young infants' behaviors such as crying, sleep and stomach issues are critical for a number of reasons: they help clinicians identify infants in need of care, help researchers identify infants at greater risk, and may help to identify new mothers who require additional support with their infant. Although the latter function of infant assessments is the least frequently used in practice, it represents a valuable addition with public health benefits to current, often observational, behavioral assessments as an implicit measure of infant-centered issues. While a number of infant behavioral assessments are currently used, there are several significant shortcomings to existing measures (e.g., restricted age of infant, requirement for extensive training of administrator, cost of the assessment, absence of established behavioral norms for comparison). Assessments of parental perception of infant behavior such as the BABI can potentially be given to large amounts of parents because they take little time and training to administer and can be used for the first half-year of an infant's life, before issues with parent-child relationships become more pervasive. Allocating resources to parents based on their perceptions of problematic infant behavior has implications for clinical practice as it could potentially help clinicians provide resources more effectively to the mother-infant dyads that need them the most.

The crying issues subscale was the only subscale in the BABI that did not have a comparative assessment where parents compared the amount that their infants cry to other infants. LF and LP were attempting to gather what they believed to be the most important indicators of domain specific infant behavior based on their expertise in infant development, and they did not believe that comparing the amount infants cry to other infants was as important as other factors such as intensity, frequency and ease of comforting. Of note, this subscale has the best internal consistency of all of the subscales (α = 0.86).

This study demonstrates the relationship between infant issues and postpartum depression, but further research is needed to tease apart the directionality of these associations. Additionally, a considerable amount of the research on infant behaviors has focused on the impact of infant crying (5, 6, 40); however, there has been very little investigation into the impact of infant stomach issues on parental psychological status and vice versa. Focusing on parent perceptions of infant behaviors could be an important area for future research for individuals interested in Public Health.

Focusing on parental perceptions of infant behavior has the potential to aid professionals such as pediatricians in triaging parent-infant dyads to closer clinical follow-up or interventional supports. Screening for postpartum depression takes place routinely at gynecological offices, primary care practices and pediatrician's offices, which serve as ideal entry points for further assessment and referral. Even though screening for postpartum depression is common in early postpartum pediatric appointments, controversy exists around whether it is intrusive to screen parents for postpartum depression and other issues at their children's health visits (rather than focusing on their child) since pediatricians are tasked with providing healthcare for the child and not necessarily the child's parents (41). To the point of focusing on the child, infants of mothers with depression and/or anxiety display variations in some behaviors [e.g., (42, 43)]. This study demonstrates the potential to focus on parental perceptions as they are related to maternal mental health and issues in parent-infant bonding.

Many of the at-home intervention programs are best understood as secondary prevention efforts and typically target populations with previously identified risk-factors [e.g., parental age, immaturity level; (44)] rather than parent report about trouble that they are having with their child. Although these programs appear to be a promising intervention for some parents, there are several limitations including parents being reluctant to having unfamiliar visitors in their homes (45), insufficient training of the individuals administering the intervention (46), and high average cost per family ($5,962) for running the programs (47). Targeting home visitations to parents with certain beliefs about their child (e.g., their infant is difficult compared to other infants), and consequently an explicit perceived need, might be more effective than targeting entire groups of parents (e.g., young parents).

A shift of focus to parent perceptions of infant behavior could help clinicians better allocate resources to the parents who need them the most. Interventions such as the Period of PURPLE Crying exist to normalize crying (48) and to prevent SBS as it relates to infant crying. The program is delivered to new parents in the hospital, in community settings such as at prenatal or well-child care visits, and through media (20). Findings are inconclusive regarding the effectiveness of the program in reducing SBS (49, 50). This might have to do with the fact that this intervention is designed to target a large audience (e.g., caregivers, community members, health care professionals) and is not specifically tailored for parents based on child characteristics such as how much they think their infant cries. We are missing critical entry points for intervention and opportunities to meet parents in the following situations: when perceived needs are high, when these concerns interact with known risk factors for maternal mental health, and when adverse outcomes occur for their offspring.

### Limitations and Future Directions

Previous studies have found infant sleep issues to be associated with maternal depression; however, the directionality of this relationship is unknown (40). That finding was not replicated in this study. Future research might consider adding sleep questions that probe perceived quality of infant sleep. Furthermore, although this study focused on risk (parent mental health and issues in parent-child bonding), a beneficial future direction will be to identify protective factors in parent-child bonding.

The cross-sectional design prevented us from looking at test-retest reliability of the BABI Scale. It also prevented us from understanding directionality in terms of infant challenging behavior and new mothers' mental health. Therefore, it is possible that maternal mental health is impacting how parents perceive their infant's behavior. Longitudinal testing will be key to determine if parent assessment of infant behavior predicts only concurrent parent psychological status or if parent assessment of infant behavior at one time point is predictive of parent psychological status at a later time point. Infant sleep issues were not related to bonding issues or mothers' reported symptoms of maternal of anxiety or depression, however, larger studies are needed to explore these issues future. Longitudinal studies should probe the directionality to better understand whether mothers of infants who exhibit stomach issues and crying are more at risk for postpartum depression or if mothers with postpartum depression are more likely to perceive their infant's behaviors to be difficult in the first place. The directionality has important implications for points of intervention. It would also be interesting to examine the impact of parent education programs on parent perception of their infant's behavior using longitudinal studies.

Additionally, the sample consists of mostly white, middle-to-upper class, educated mother-child dyads, which may limit the generalizability to other diverse social and cultural groups. Researchers should attempt to replicate study findings with larger more diverse samples, and information about the test-retest reliability of this measure over time needs to be gathered. It is possible that relationships between infant behaviors such as sleep and parent mental health and infant-parent bonding issues will be significant with larger samples. Therefore, researchers should continue to pursue these research questions with larger more diverse samples. Additionally, further analyses can be done to examine if parent perceptions of infant behavior differ across parents from different demographic backgrounds. It would also be interesting to examine trends in parent perceptions of infant behavior across first-time vs. experienced parents.

Assessing parent perceptions of challenging infant behaviors represents a potential way for health professionals to identify infants who are higher risk and in need of intervention and parents in need of additional support. It may be of particular clinical importance for researchers to establish cutoff scores in order to screen for parents who indicate higher risk for problems such as bonding issues with their infant, infant abuse or parental depression and anxiety. However, these scores can only be determined through the application of this scale with large, diverse samples of parents of newborns.

## Data Availability Statement

The raw data supporting the conclusions of this article will be made available by the authors, without undue reservation.

## Ethics Statement

The studies involving human participants were reviewed and approved by University of Houston IRB. The patients/participants provided their informed consent to participate in this study.

## Author Contributions

LF is first author and TU is responsible for statistical analysis. All authors contributed to and approve the manuscript.

## Funding

The research in this publication was supported by the Provost's 50-in-5 award at the University of Houston. The content is solely the responsibility of the authors and does not necessarily represent the official views of the University of Houston.

## Conflict of Interest

The authors declare that the research was conducted in the absence of any commercial or financial relationships that could be construed as a potential conflict of interest.

## Publisher's Note

All claims expressed in this article are solely those of the authors and do not necessarily represent those of their affiliated organizations, or those of the publisher, the editors and the reviewers. Any product that may be evaluated in this article, or claim that may be made by its manufacturer, is not guaranteed or endorsed by the publisher.
